# Agomelatine augmentation of sertraline in the treatment of moderate to severe obsessive-compulsive disorder: a randomized double-blinded placebo-controlled clinical trial

**DOI:** 10.1186/s12888-023-05189-7

**Published:** 2023-09-21

**Authors:** Marjan Shokrani, Sanaz Askari, Negin Eissazade, Seyed Vahid Shariat, Behnam Shariati, Masoomeh Yarahmadi, Mohammadreza Shalbafan

**Affiliations:** 1 Mental Health Research Center, Psychosocial Health Research Institute (PHRI), Department of Psychiatry, School of Medicine, Iran University of Medical Sciences, Tehran, Iran; 2 Student Research Committee, School of Medicine, Iran University of Medical Sciences, Tehran, Iran; 3 Brain and Cognition Clinic, Institute for Cognitive Sciences Studies, Tehran, Iran

**Keywords:** Agomelatine, Obsessive-compulsive disorder, Sertraline, Selective serotonin reuptake inhibitors

## Abstract

**Background:**

As 40–60% of the patients with obsessive-compulsive disorder (OCD) do not adequately respond to the first-line treatment, finding an effective second-line treatment is required. Our aim was to assess the efficacy and safety of agomelatine (a selective melatonin receptor agonist and a 5-hydroxytryptamine (HT)2 C antagonist) augmentation of sertraline in the treatment of patients with moderate to severe OCD.

**Methods:**

In this 12-week randomized, double-blinded, placebo-controlled, parallel-group clinical trial, 65 patients with moderate to severe OCD according to the Diagnostic and Statistical Manual of Mental Disorders-Fifth edition (DSM–5) criteria and a Yale-Brown obsessive compulsive scale (Y-BOCS) score of over 21, were included. They were assigned with sertraline (100 mg/day for the first 4 weeks and 200 mg/day for the next 8 weeks) and either agomelatine (25 mg/day) or placebo. The primary outcome was OCD symptoms measured by the Y-BOCS.

**Results:**

Fifty patients (24 in agomelatine group and 26 in placebo group) completed the trial. The Y-BOCS scores in total (MD (95% CI) = 12.25 (11.00, 13.49) (P < 0.001) vs. MD (95% CI) = 12.46 (6.65, 15.74) (P < 0.001)), the obsession subscale (MD (95% CI) = 5.04 (4.19, 5.88) (P < 0.001) vs. MD (95% CI) = 5.00 (3.84, 6.16) (P = 0.0001)), and compulsion subscale (MD (95% CI) = 7.21 (6.34, 8.07) (P < 0.001) vs. MD (95% CI) = 7.460 (6.50, 8.42) (P < 0.001)) significantly decreased in both groups. Although, at the end of the trial, no significant difference was observed between the scores of the two groups in total (MD (95% CI) = 0.480 (-1.23, 2.19) (P = 0.78)), the obsession subscale (MD (95% CI) = 1.020 (-0.15, 2.19) (P = 0.38)), and the compulsion subscale (MD (95% CI) = 0.540 (-0.34, 1.42) (P = 0.54)). No major adverse effects were recorded, and the frequency of side effects was not significantly different between the groups.

**Conclusion:**

Agomelatine in augmentation with sertraline is safe and tolerable in patients with moderate to severe OCD. However, our study does not support its efficacy in improving OCD symptoms, compared to placebo.

**Trial registration:**

The trial was registered at the Iranian Registry of Clinical Trials on 14/07/2020 (www.irct.ir; IRCT ID: IRCT20170123032145N5).

## Background

Obsessive-compulsive disorder (OCD) is a chronic mental disorder characterized by obsessions, compulsions, or both. It presents with recurring, unwanted, and intrusive thoughts, images, or urges (obsessions) that may lead to repetitive behaviors and mental acts suppressing the distress caused by obsessive thoughts (compulsions). OCD is one of the most common psychiatric disorders, and its lifetime prevalence is 2.3% among the general population [1–3]. It causes clinically significant impairment in critical areas of functioning [3], and as the World Health Organization (WHO) stated, it is one of the ten most disabling conditions [4].

Currently, the first-line treatment of OCD includes selective serotonin reuptake inhibitors (SSRIs), clomipramine, and cognitive-behavioral therapy (CBT) [5, 6]. With 40–60% of the patients being treatment-refractory, searching for efficacious second-line treatment is required [7, 8].

The pathophysiology of OCD is not yet fully understood. Although, hormonal dysregulation and delayed sleep phase disorder (DSPD) in patients with OCD point to the possible role of abnormal circadian rhythms [9]. Moreover, it has been reported that more severe OCD symptoms are associated with more significant sleep disturbance [10]. Melatonin, a serotonin product, is released from the pineal gland and plays a key role in regulating the circadian rhythm. It has been reported that in patients with OCD, the night-time peak of melatonin is significantly reduced and delayed for two hours [11, 12], which leads to a phase delay in sleep. Also, the increase in the nocturnal secretion of adrenocorticotropic hormone (ACTH) and corticotropin-releasing hormone (CRH) suggests an increase in the activity of the hypothalamic-pituitary-adrenal (HPA) axis [13, 14]. Sleep disturbance affects mood, reward-related brain activity, and neurobehavioral function [15]. Nevertheless, proxy measurement of the circadian rhythm did not back up the association between melatonin and OCD [14], and the role of circadian rhythm in the pathophysiology of OCD is still unclear.

Agomelatine is a selective melatonin receptor (MT_1_ and MT_2_) agonist and a 5-hydroxytryptamine-2 C (5-HT_2C_) antagonist. It is a sleep-modulating antidepressant that promotes neurogenesis as well. It has been approved by European Medicines Evaluation Agency (EMEA) for treating major depressive disorder (MDD). Although, it is associated with high risks of acute liver injury [16]. Through its melatonergic pathway, agomelatine resynchronizes the circadian rhythm and improves reward mechanism and incentive motivation. Predominantly through the blockade of 5-HT2C, it disinhibits the norepinephrine and dopamine firing, leading to increased levels of dopamine and noradrenaline in the frontal cortex, and reduced stress-induced increase of glutamate [17–21]. These theories have put forward the potential beneficence of agomelatine in the treatment of patients with OCD, and a few studies [22–26] have evaluated its efficacy and safety.

We conducted this randomized, double-blinded, placebo-controlled, parallel-group clinical trial to assess the efficacy and safety of agomelatine augmentation of sertraline in the treatment of patients with moderate to severe OCD.

## Methods

This 12-week randomized, double-blinded, placebo-controlled, parallel-group clinical trial was conducted in the out-patient clinics of (1) Iran Psychiatric Hospital, (2) Rasool-Akram Hospital, and (3) Tehran Institute of Psychiatry, and (4) the Brain and Cognition Clinic (affiliated with Iran University of Medical Sciences, Tehran, Iran) from April to November 2022.

Participants enrolled in the study were randomized using the block method (allocation ratio 1:1, blocks of four). Treatment allocation concealment was ensured by using sequentially numbered, opaque sealed envelopes. Participants, outcome assessors, and the statistical analyst were separate individuals blinded to allocation. Additionally, placebos had identically matched size, shape, color, odor, and pharmaceutical packaging with the agomelatine tablets.

Participants were men and women, aged between 18 and 60 years, diagnosed with obsessive-compulsive disorder based on the Diagnostic and Statistical Manual of Mental Disorders, 5th Edition (DSM-5) criteria [27]. All patients were assessed by a board-certified psychiatrist using a structured clinical interview designed based on the DSM-5 criteria, and those with a Yale–Brown Obsessive Compulsive Scale (Y-BOCS) [28] score of ≥ 21 (moderate to severe OCD) were included. The exclusion criteria were: (1) having received any psychiatric treatment over the past six weeks, (2) life-threatening psychiatric conditions (e.g., suicidal thoughts), (3) comorbid any other psychiatric disorder (e.g., schizophrenia and bipolar disorder), (4) comorbid severe medical (e.g., neurological, hepatic, and cardiac) conditions, (5) intellectual disability (based on clinical judgment), (6) substance use disorders (except for nicotine), (7) pregnancy or breastfeeding, (8) contraindications for agomelatine or sertraline, (9) history of complete response to sertraline, (10) history of OCD-related psychosurgery, and (11) treatment-refractory OCD. Participants did not receive any psychotherapeutic treatment during the trial.

The parallel groups were randomly assigned with sertraline 200 mg/day and either agomelatine 25 mg/day or placebo for 12 weeks. Sertraline was started at 25 mg/day and if tolerable, up-titrated to 100 mg/day for the first four weeks, and 200 mg/day for the next eight weeks.

We used the Persian version of Y-BOCS [29–34] to assess the patients at baseline and the fourth, eighth, and twelfth weeks of the study. Y-BOCS is a 10-item semi-structured interview administered by a trained clinician to determine the type and the severity of OCD symptoms over the past seven days (from 0 to 4) [35].

The primary outcome measure was the mean difference between the Y-BOCS total scores between the two groups from baseline to the twelfth week. The secondary outcome measures were (1 and 2) mean differences between the Y-BOCS obsession and compulsion subscales scores between the two groups from baseline to the twelfth week, and (3) frequency and severity of adverse effects in the two groups.

We monitored the treatment-related adverse effects during every patient visit. This comprehensive evaluation involved both patient self-reports and careful observations by the attending psychiatrist. If a medication became intolerable or posed a life-threatening risk to the patient, resulting in the discontinuation of treatment, it was categorized as severe. All other adverse effects were categorized as non-severe. It was agreed that in case of insomnia, oxazepam 10 mg, and in case of tremor or agitation [36], propranolol 10 mg should be prescribed [37]. To avoid the risk of hepatotoxic reactions of agomelatine [19], the LFTs (liver function tests), including alanine transaminase (ALT), aspartate aminotransferase (AST), and alkaline phosphatase (ALP), were measured at baseline and the endpoint of the study for all of the patients. In case of detecting more than three-fold increases, discontinuation of the medication and undergoing further evaluation were indicated.

By using a between-groups difference of 5 in the Y-BOCS score, a 95% confidence interval, a power of 80%, and an attrition rate of 20%, a sample size of 44 (22 in each group) was calculated with G-power 3.1.9.2 [27]. To retain the original randomization of the patients, the last observation carried forward (intent-to-treat) method was used.

Continuous variables are presented as mean ± standard deviation. Categorical variables were analyzed by Pearson’s Chi-squared test and Fisher’s exact test. The mean differences of the Y-BOCS scores among each group were calculated using the paired sample t-test. The mean differences of the Y-BOCS scores between the groups were calculated using the Independent T-test. Cohen’s d effect sizes were calculated. Group-by-time interaction effect was evaluated using the repeated measures ANOVA, through a general linear model algorithm. Cohen’s d effect sizes were calculated. A p-value of 0.05 or less was considered statistically significant. All statistical analyses were performed with the Statistical Package for the Social Sciences (SPSS) software for Windows (version 24, SPSS Inc., Chicago, IL, USA).

## Results

**Fig. 1:**
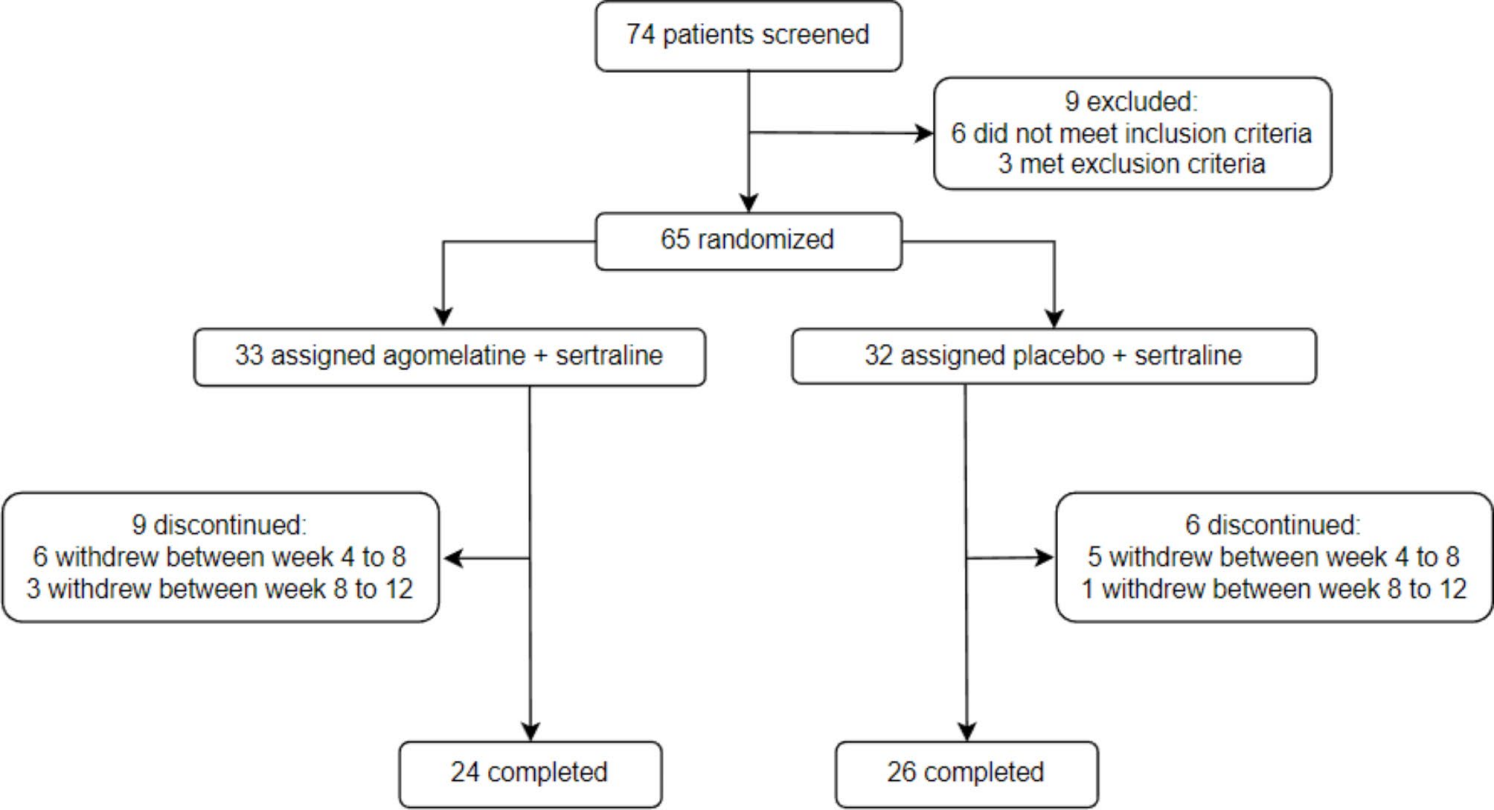
Flowchart of the participants enrolled in the trial

Out of the 74 screened patients, 65 were recruited and randomly assigned to sertraline and agomelatine (N = 33) or sertraline and placebo (N = 32) groups. As presented in Figs. 1 and 15 patients withdrew from the study due to personal reasons, and 50 patients completed the trial (32 female, and 18 male). The mean (± SD) age of the participants was 37.22 (± 12.181). Demographic characteristics of the participants are presented in Table 1.

**Table 1:** Demographic characteristics of the participants

		Agomelatine + sertraline (n = 24)		Placebo + sertraline (n = 26)	
		**Mean (± SD)**	**Count (%)**	**Mean (± SD)**	**Count (%)**
Age (years)		37.79 (± 12.78)		36.69 (± 11.82)	
Gender	**Female**		15 (62.50%)		17 (65.38%)
	**Male**		9 (37.50%)		9 (34.61%)
Education	**Middle school**		-		1 (3.84%)
	**High school diploma**		6 (25%)		4 (15.38%)
	**Associate’s degree**		2 (8.33%)		3 (11.53%)
	**Bachelor’s degree**		11 (45.83%)		11 (42.30%)
	**Master’s Degree**		5 (20.83%)		6 (23.07%)
	**PhD**		-		1 (3.84%)
Marital status	**Single**		9 (37.50%)		10 (38.46%)
	**Married**		15 (62.50%)		16 (61.53%)
Employment	**Employed**		14 (58.33%)		15 (57.69%)
	**Unemployed**		3 (12.50%)		5 (19.23%)
	**Housewife**		6 (25%)		4 (15.38%)
	**Student**		1 (4.16%)		2 (7.69%)

The baseline Y-BOCS scores were not significantly different between the groups in total (MD (95% CI) = 0.270 (-1.062, 1.602) (P = 0.8403)), the obsession subscale (MD (95% CI) = 1.060 (0.204, 1.916,) (P = 0.2214)) and compulsion subscale (MD (95% CI) = 0.790 (-0.168, 1.748) (P = 0.4136)) (Table 2).

**Table 2:** Baseline Y-BOCS scores of the patients

	Agomelatine + sertraline (n = 24)	Placebo + sertraline (n = 26)	MD (95% CI)	P-value
Obsession	14.33 ± 2.426	13.27 ± 3.482	1.060 ± 0.856	0.2214
Compulsion	12.71 ± 3.155	13.50 ± 3.581	0.790 ± 0.958	0.4136
Total	27.04 ± 4.457	26.77 ± 4.926	0.270 ± 1.332	0.8403

**Fig. 2:**
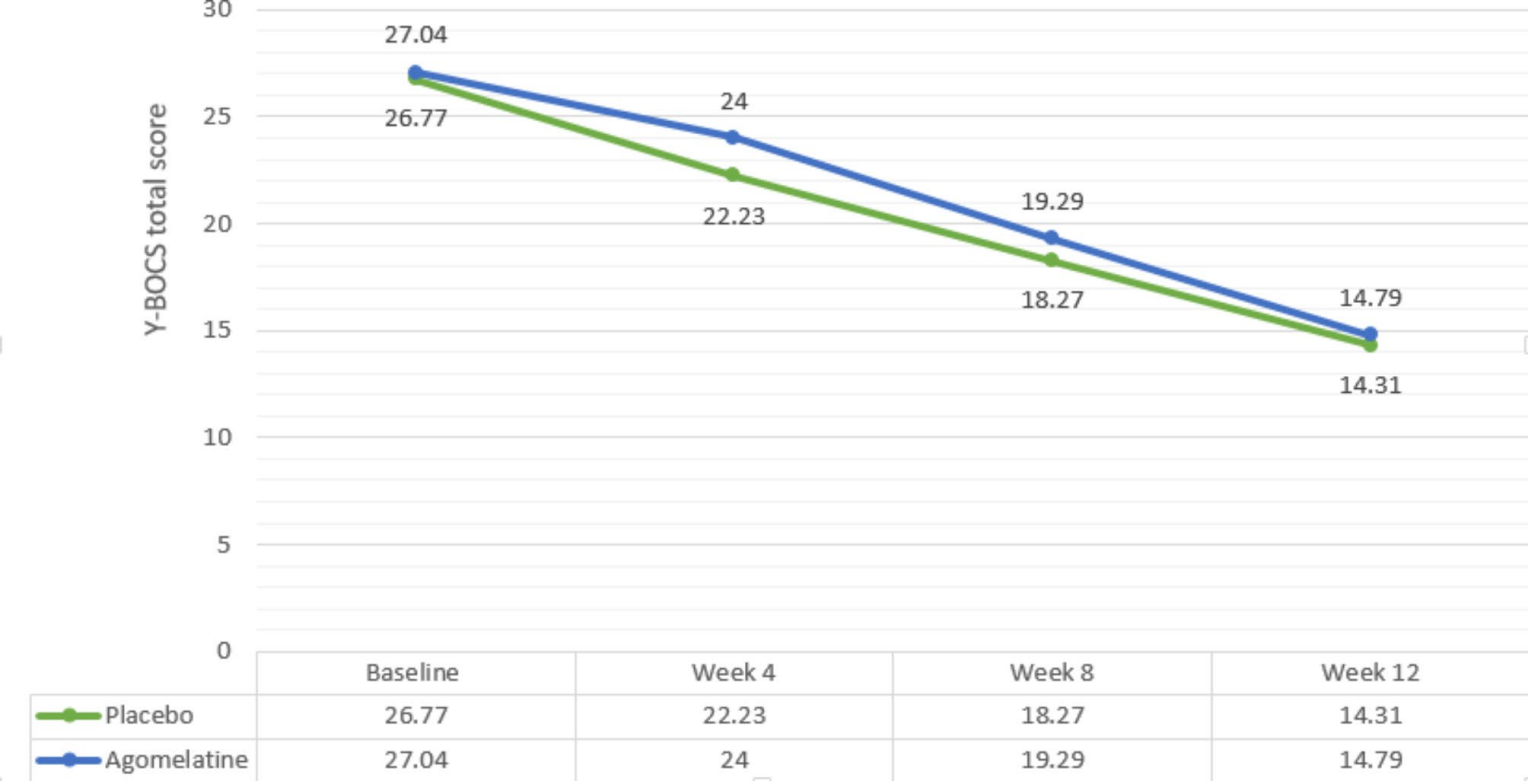
Comparison of the total Y-BOCS score changes between the two groups during the course of trial

Total Y-BOCS scores significantly dropped in both groups, slightly more in the placebo group. The score changes from baseline in the agomelatine group at weeks 4, 8 and 12 were MD (95% CI) = 3.04 (1.63, 4.44) (P = 0.03), MD (95% CI) = 7.75 (6.49, 9.00) (P < 0.001), and MD (95% CI) = 12.25 (11.00, 13.49) (P < 0.001), respectively. Furthermore, score changes from baseline in the placebo group at weeks 4, 8, and 12 were MD (95% CI) = 4.54 (1.21, 9.98) (P = 0.03), MD (95% CI) = 8.50 (6.65, 4.443) (P < 0.001), and MD (95% CI) = 12.46 (6.65, 15.74) (P < 0.001), respectively (Fig. 2) (Table 3).

The mean difference between the two groups at weeks four (MD (95% CI) = 1.770 (1.417) (P = 0.21)), eight (MD (95% CI) = 1.020 (1.413) (P = 0.47)) and twelve (MD (95% CI) = 0.480 (-1.23, 2.19) (P = 0.78)) were not statistically significant. Also, repeated-measures analysis did not reveal a significant effect of time (Greenhouse-Geisser F (2.176, 104.426) = 0.546, P = 0.59).

**Table 3:** The mean (± standard deviation) Y-BOCS scores of weeks 4, 8, and 12, and their comparison with the baseline scores (95% CI)

		Agomelatine + sertraline (n = 24)				Placebo + sertraline (n = 26)			
		**Mean ± SD**	**MD**	**P-value**	**Cohen’s d effect size**	**Mean ± SD**	**MD**	**P-value**	**Cohen’s d effect size**
Obsession	**Week 4**	13.4 ± 2.76	0.91 ± 0.7	0.2	0.29	11.4 ± 3.8	1.8 ± 1.01	0.08	0.48
	**Week 8**	11.4 ± 2.51	2.91 ± 0.7	< 0.001	0.89	9.9 ± 3.95	3.35 ± 1.03	0.002	0.89
	**Week 12**	9.3 ± 3.32	5.04 ± 0.8	< 0.001	1.33	8.3 ± 4.8	5 ± 1.16	< 0.001	1.19
Compulsion	**Week 4**	10.6 ± 4.05	2.13 ± 1	0.05	0.68	10.8 ± 3.14	2.73 ± 0.93	0.005	0.81
	**Week 8**	7.9 ± 3.01	4.83 ± 0.9	< 0.001	1.51	8.3 ± 3.22	5.15 ± 0.94	< 0.001	1.51
	**Week 12**	5.5 ± 2.84	7.21 ± 0.9	< 0.001	2.22	6 ± 3.34	7.46 ± 0.96	< 0.001	2.15
Total	**Week 4**	24 ± 5.23	3.04 ± 1.4	0.03	0.66	22.2 ± 4.8	4.54 ± 1.34	0.001	0.93
	**Week 8**	19.3 ± 4.25	7.750 ± 1.2	< 0.001	1.53	18.3 ± 5.6	8.5 ± 1.46	< 0.001	1.61
	**Week 12**	14.8 ± 4.18	12.25 ± 1.2	< 0.001	2.01	14.3 ± 7.4	12.46 ± 1.74	< 0.001	1.98

**Fig. 3:**
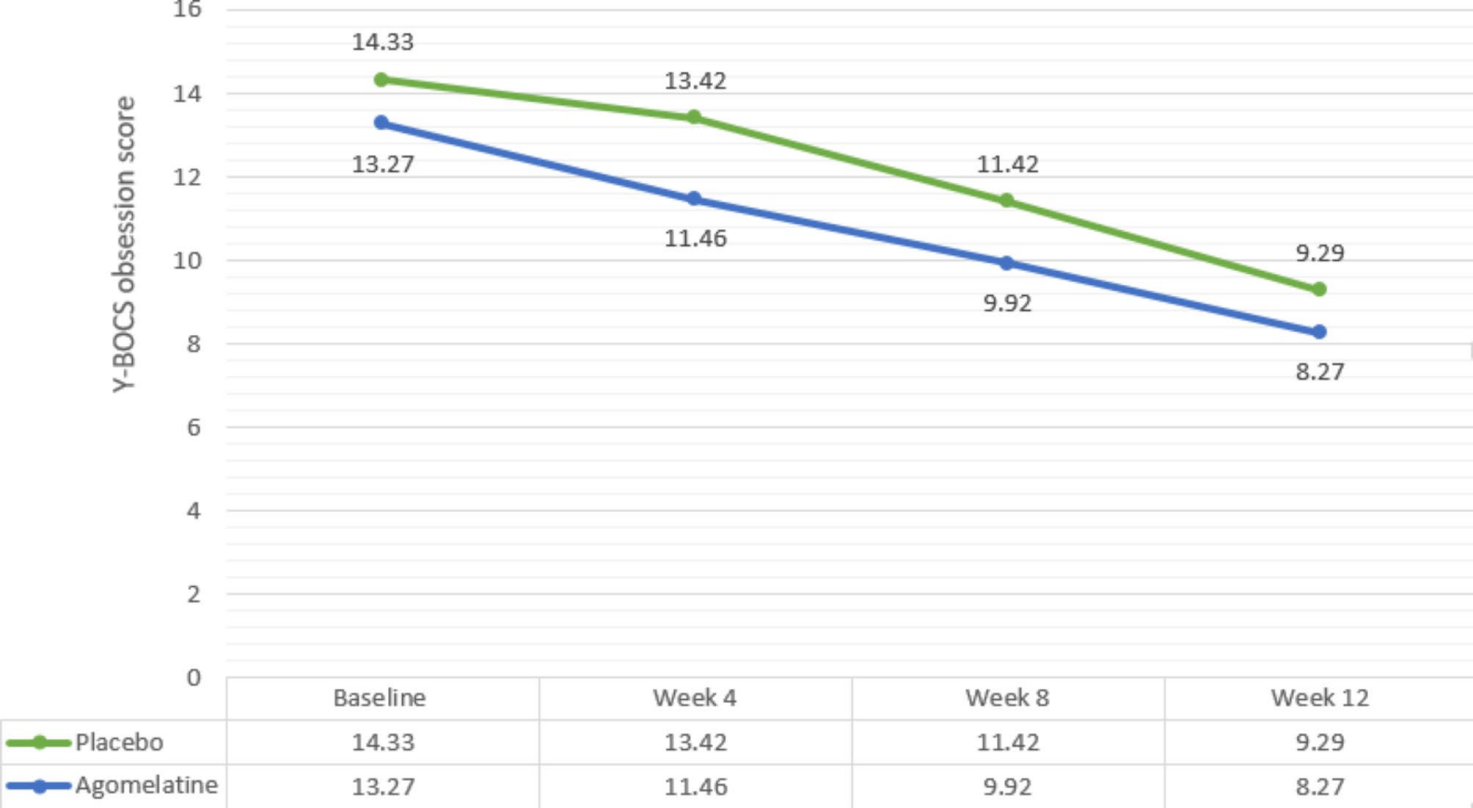
Comparison of Y-BOCS score changes between the two groups during the course of trial

Y-BOCS obsession subscale score changes from baseline in the agomelatine group at weeks 4, 8 and 12 were MD (95% CI) = 0.91 (0.15, 1.66) (P = 0.23), MD (95% CI) = 2.91 (2.19, 3.66) (P = 0.0002), and MD (95% CI) = 5.04 (4.19, 5.88) (P < 0.001), respectively. Score changes from baseline in the placebo group at weeks 4, 8 and 12 were MD (95% CI) = 1.81 (0.79, 2.8) (P = 0.07), MD (95% CI) = 3.35 (2.31, 4.38) (P = 0.002), and MD (95% CI) = 5.00 (3.84, 6.16) (P < 0.001), respectively (Fig. 3) (Table 3).

At week 4, the Y-BOCS scores significantly dropped in the placebo group (MD (95% CI) = 1.960 (0.948) P = 0.04However, the mean differenced at weeks eight (MD (95% CI) = 1.500 (0.947) (P = 0.11)) and twelve (MD (95% CI) = 1.020 (-0.15, 2.19) (P = 0.38)) were not statistically significant (Fig. 3) (Table 3). Repeated measure ANOVA analysis did not detect a significant Time X Treatment interaction (Greenhouse-Geisser F (1.965, 94.315) = 0.576, P = 0.56).

**Fig. 4:**
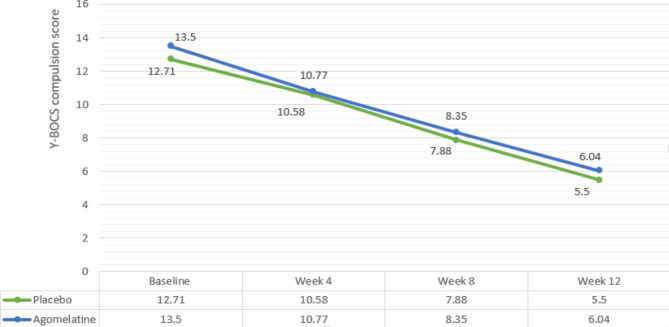
Comparison of Y-BOCS score changes between the two groups during the course of trial

The Y-BOCS compulsion subscale score changes from baseline at weeks 4, 8 and 12 of the study in the agomelatine group were MD (95% CI) = 2.13 (1.08, 3.17) (P = 0.04), MD (95% CI) = 4.83 (3.94, 5.72) (P < 0.0001), and MD (95% CI) = 7.21 (6.34, 8.07) (P < 0.001), respectively. And in the placebo group, the score changes from baseline at weeks 4, 8 and 12 were MD (95% CI) = 2.73 (1.79, 3.66) (P = 0.005), MD (95% CI) = 5.15 (4.20, 6.09) (P < 0.001), and MD (95% CI) = 7.460 (6.50, 8.42) (p-value < 0.001), respectively (Fig. 4) (Table 3).

The mean difference between the two groups at weeks four (MD (95% CI) = 0.190 (1.021) (p-value = 0.85)), eight (MD (95% CI) = 0.470 (0.884) (p-value = 0.59)) and twelve (MD (95% CI) = 0.540 (-0.34, 1.42) (P = 0.54)) were not significantly different (Table 3).

Additionally, no significant difference was detected via repeated-measures analysis (Huyn-Feldt F (2.394, 114.931) = 0.165, P = 0.88).

High rates of LFTs were not detected in any of the patients.

Adverse events were recorded during the study and were characterized as transient and non-severe, with no cases necessitating treatment discontinuation. The frequency of side effects was not significantly different between the groups (Table 4). Oxazepam 5 mg was given to 3 patients in the agomelatine group and 2 in the placebo group due to insomnia.

**Table 4:** Comparison of the frequency of adverse events between the two groups (N, %)

	Agomelatine + sertraline (n = 24)	Placebo + sertraline (n = 26)	P-value
Insomnia	2 (8.3%)	3 (11.5%)	0.57
Agitation	1 (4.1%)	2 (7.6%)	0.60
Dry mouth	1 (4.1%)	1 (3.8%)	0.95
Sedation	2 (8.3%)	2 (7.6%)	0.93
Constipation	-	1 (3.8%)	0.33

## Discussion

We did not find a significant difference between agomelatine and placebo in augmentation with sertraline, in improving the symptoms of moderate to severe OCD. The observed adverse effects were mild and did not cause withdrawal. Furthermore, the frequency and severity of adverse effects were not notably different between the two groups.

Our results tie well with another clinical trial [22], a 16-week randomized, double-blind placebo-controlled phase II study that included 72 patients (39 on agomelatine and 35 on placebo) with moderate to severe OCD. Unlike our study, agomelatine 25 mg/day was used as mono-therapy (50 mg/day in case of ≤ 20% reduction of the Y-BOCS total score at week eight). On the other hand, four other studies reached a different conclusion. However, none of them were clinical trials (high risk of bias), and had included patients with treatment-resistant OCD. Additionally, they all did not augment agomelatine with the sertraline: (1) E Tzavellas et al. (open-label case series) reported that agomelatine, combined with a SSRI, reduced the Y-BOCS score by 25% on average, in 12 patients [23], (2) Fornaro (case series) reported that agomelatine (50 mg/day) helped with the improvement of OCD symptoms in 3 out of 6 SRI-refractory patients. Patients with comorbid mood disorders were not excluded from the study [24]. (3) da Rocha FF et al. (case report) reported that their patient clinically improved after taking agomelatine (25 mg/day) in augmentation with clomipramine (225 mg/day) [25], and (4) De Berardis D et al. (case report) reported that their patient remitted (Y-BOCS score decreased from 33 to 6) after five weeks of taking agomelatine (25 mg/day) combined with escitalopram (30 mg/day) [26].

Similar to our study, all the previous studies [22–26] have stated that agomelatine is well-tolerated and causes no major side effects. The side effects observed in the agomelatine group of our study were insomnia, sedation, and dry mouth. The previously reported side effects of agomelatine include nausea, headache, dizziness, weight gain, and somnolence [27–30].

None of the patients included in our study suffered from substantial sleep disturbance at the initial evaluation, and the incidence of insomnia was not significantly different between the groups. However, a clinical trial [22] and a case report [26] reported that their patients experienced an improvement in their sleep quality. DSPD and abnormal circadian rhythms affect mood, physical and mental well-being, leading to poor daily-life performance. Moreover, sleep disturbance increases the risk of developing MDD and schizophrenia, and late-life suicid [38, 39]. Therefore, improving the sleep quality of patients with OCD not only improves their life quality but also helps prevent the worsening of their condition.

The exact OCD-related mechanism of action and clinical effects of agomelatine are still unclear, but it has been shown to be effective in the resynchronization of circadian rhythms, selectively enhancing the frontocortical dopaminergic pathway, enhancing the frontocortical adrenergic pathway, promotion of hippocampal neurogenesis (by increasing brain-derived neurotrophic factor (BDNF)), and increasing the activity of hypothalamic–pituitary–adrenal (HPA) axis [13, 14]. The preceding statements propose the potential therapeutic effect of agomelatine on OCD. Nevertheless, we did not find any marked improvement in our patients’ symptoms.

### Limitations

Our study was limited by small sample size, short duration, not including patients with comorbidities, and high attrition rate (23.07%). Additionally, we only included patients with moderate to severe OCD, and our results are not generalizable to patients with treatment-resistant OCD. Further multi-center clinical trials with extended follow‑up period and large sample size are needed to assess the effect of agomelatine on OCD symptoms. We also suggest including patients with treatment-resistant OCD, sleep disturbance and other comorbidities.

## Conclusion

Agomelatine in augmentation with sertraline is safe and tolerable, but in comparison with placebo, it does not significantly improve the symptoms of OCD. Further research is needed to confirm the findings.

## Data Availability

The datasets generated and/or analyzed during the current study are not publicly available due to confidentiality concerns (in the informed consent form, we made a commitment to the participants to publish only the general and group results of the study) but are available from the corresponding author on reasonable request.

## Abbreviations

OCD: Obsessive-Compulsive Disorder
SSRI: Selective Serotonin Reuptake Inhibitor
CBT: Cognitive Behavioral Therapy
DSM–5: Diagnostic and Statistical Manual of Mental Disorders-5th Edition
DSPD: Delayed Sleep Phase Disorder
ACTH: Adrenocorticotropic Hormone
CRH: Corticotropin-Releasing Hormone
MDD: Major Depressive Disorder
Y-BOCS: Yale-Brown Obsessive Compulsive Scale
RCT: Randomized Controlled Trial
MD: Mean Difference
CI: Confidence Interval
SD: Standard Deviation
